# The Proportion of Complementary and Alternative Medicine Utilization Among Saudi Population for Eye Care: Cross-Sectional Study

**DOI:** 10.7759/cureus.13109

**Published:** 2021-02-03

**Authors:** Salma AlSalman, Munira A AlHussaini, Rajiv B Khandekar, Deepak P Edward

**Affiliations:** 1 Ophthalmology, King Khaled Eye Specialist Hospital, Riyadh, SAU; 2 Ophthalmology, College of Medicine, King Saud Medical City, Riyadh, SAU; 3 Ophthalmology, King Khalid Eye Specialist Hospital, Riyadh, SAU; 4 Ophthalmology, Faculty of Medicine, University of British Columbia, Vancouver, CAN; 5 Ophthalmology and Visual Sciences, University of Illinois College of Medicine, Chicago, USA

**Keywords:** traditional medicine, eye, ophthalmic treatment

## Abstract

Purpose

To estimate the prevalence, determinants and perceived benefit of complementary and alternative medicine (CAM) use for ophthalmic purposes among the Saudi population.

Methods

A cross-sectional study was carried out in 2019 targeting visitors of King Khaled Eye Specialist Hospital (KKESH), Riyadh, Saudi Arabia. The survey had 18 questions in Arabic regarding the use of CAM for eye ailments. The magnitude was associated to the demographic and eye-related factors.

Results

We interviewed 999 participants. The prevalence of CAM usage was 21.9% (95% confidence interval 19.3; 24.5). Castor oil (49.3%), antimony (khohl alethmed) (40.6%), chamomile (19.6%), and green tea leaves (11.4%) were the most common medications used. Other traditional eye treatments included eye vitamins, faith healing (prayer, reciting Quran, Zamzam water), cautery, cupping, and acupuncture.

Conclusions

One in five Saudi eye patients used CAM. The factors governing this health behavior should be studied to change this practice pattern.

## Introduction

Worldwide, the last two decades have witnessed a phenomenal increase in the prevalence of the use of complementary and alternative medicine (CAM) [1,2]. CAM is defined by the National Center for Complementary and Integrative Health as “… complementary medicine as the use of a non-mainstream practice together with conventional medicine and alternative medicine as using non-mainstream practices in place of conventional medicine" [3]. The use of CAM can be for acute or chronic disease, as a prevention or treatment modality [4].

Universal interest in the use of CAM ranging between 9% and 70% of the population, although sufficient scientific evidence for its use is lacking [5-7]. Regionally, the prevalence of CAM use varies from 21.6% to 90.5% [8]. CAM practice varies widely between countries depending on their tradition and disease prevalence. In Saudi Arabia, the practice of CAM is common and used in many fields. Using honey, black seed, myrrh and alhijama (cupping) are considered to be of special therapeutic value in our region due to religious beliefs. It has been reported that nearly half of Saudi patients have visited a traditional healer [9,10]. Several other studies addressed the use of CAM with dermatological diseases, cancer, psychiatric disorders and liver diseases which showed a prevalence of 40%, 55%, 74.1%, and 90%, respectively [9-12]. Although there are several reports on the use of CAM in multiple fields, there is limited data to address the frequency of CAM utilization with ophthalmological diseases.

Local case reports revealed ocular adverse effects including severe acute ocular injuries after using kermes, and ocular chemical burn after using the seeds of Lepidium sativum (Rashad) [13,14]. It is important for clinicians to be aware of the significant potential adverse effects that have been associated with nutritional supplements and herbal medications and to question patients regarding their use, as patients frequently do not disclose this information to their physicians. Shedding light on commonly used CAM in ophthalmology can provide data on the extent CAM is used in the country and can help in improving physician knowledge about the use and determine lines of questioning during history taking and understanding drug-CAM interaction.

In our review of the literature, we have not found any existing data on the frequency and types of CAM utilization among Saudi patients with ophthalmological diseases. The aim of this study is to estimate the prevalence of complementary and alternative medicine use for ophthalmological disease, determine the types and nature of CAM, explore possible demographic and disease-related associations, and inquire about the perceived benefit of these treatments.

## Materials and methods

This was a cross-sectional survey. The institutional research board of King Khaled Eye Specialist Hospital (KKESH approved of this study (P 1805). Informed written consent was obtained from each participant. Eye patients and their relative visiting the outpatient department of our institution from January to June 2018 were our study population. Adults aged 18 to 90 years and consenting to participate in the survey were included in this study. All tenets of the Helsinki declaration were strictly followed in the study.

To calculate the sample size for the study we assumed that the use of CAM among the Saudi population is 25.7% [15]. For a population of 30,000 that attended annual our hospital, to achieve a 95% confidence interval, 5% acceptable error margin with the design effect “2” we need to randomly select at least 582-600 Saudi adults to be interviewed. This calculation was done using OpenEpi software [16].

Data were collected using interviewer-administered questionnaire. The questionnaire in Arabic was either given to the participant to fill out under the supervision of the investigator or was filled out by the investigator while interviewing the participant. The agreement rate of the two co-investigators methodology after a standardized training session was 85% during the pilot. The survey development process included the following phases: review of existing literature, question drafting and finalization of written questions. The validity of questionnaire using Pearson Product Moment correlation was <0.005 in each English and Arabic questionnaire. While testing for the reliability of survey tool, the Cronbach's Alpha value was 0.72 suggesting high reliability. The demographic information included age, gender, location of resident in relation to the region of Saudi Arabia, education level and occupation of the participant. The questionnaire also focused on general health-related information Data on CAM covered the products they use, for ophthalmic benefits or for other health reasons, source of knowledge about the products, with whom they discussed their use and the perceived benefit of CAM. The survey questions avoided open-ended questions and double questions. Participants were also allowed to mention additional CAM they used.

**Figure 1:**
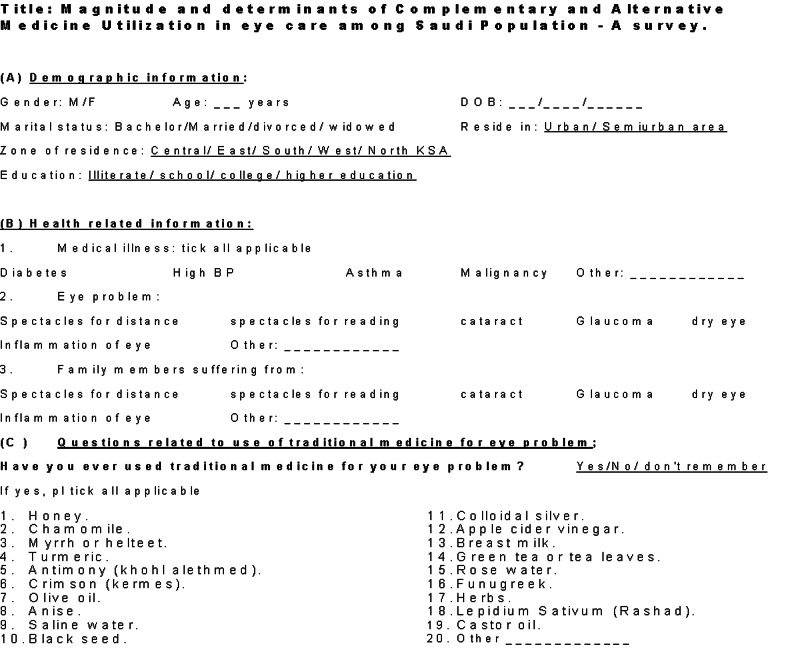
Appendix page 1 data collection form.

**Figure 2:**
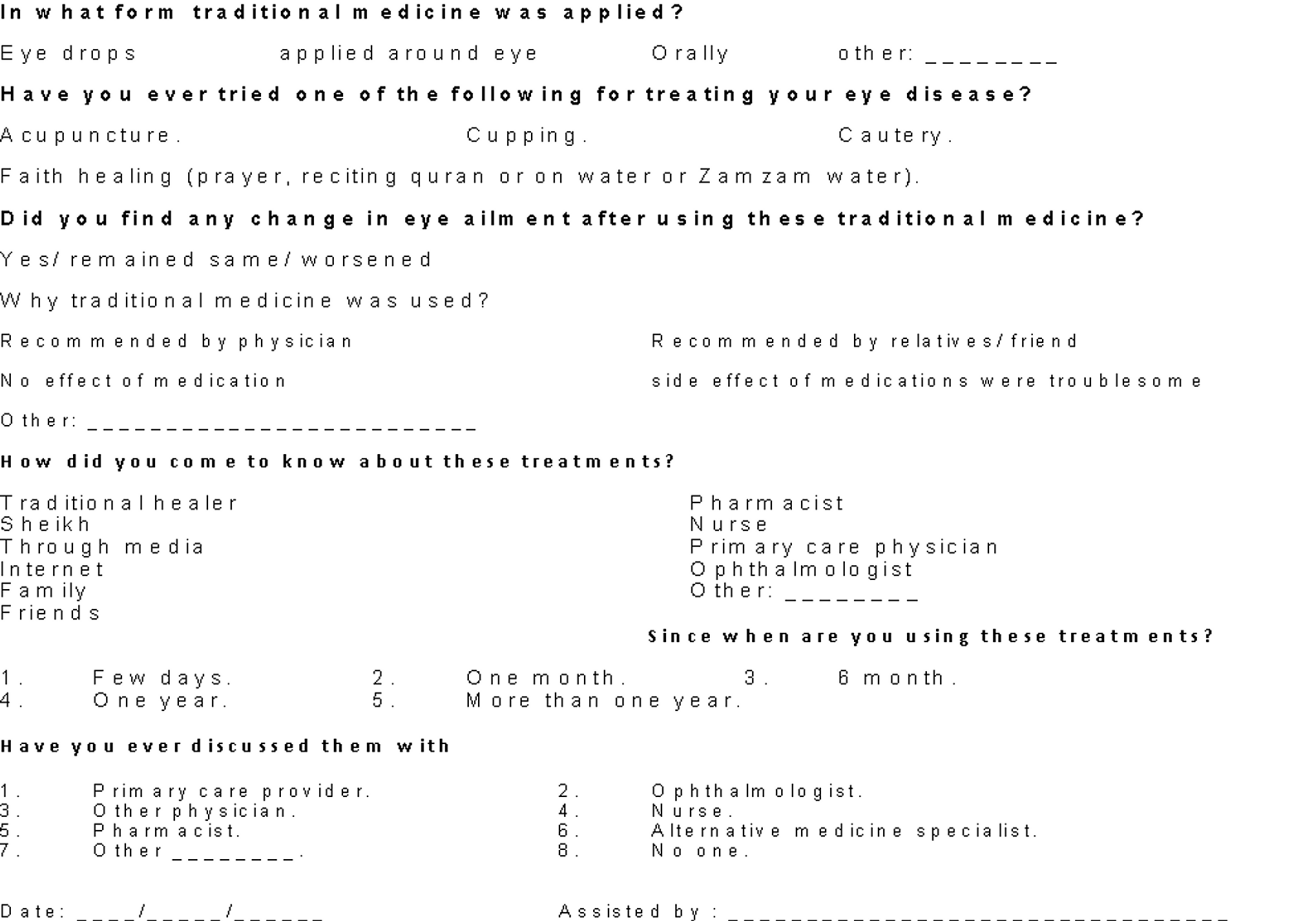
Appendix 1 page 2 data collection form.

The data were collected using pre-tested data collection form (Figures 1, 2: appendix). Then It was then transferred to the Microsoft excel® spreadsheet. A panel of three experts responded to the questionnaire and based on their consensus, the correct response of the participant was determined. For data management and analysis, the Statistical Package for Social Sciences (SPSS 25) (IBM Corp., Armonk, NY). Descriptive analyses were carried out by computing the frequencies and percentages for categorical variables. The prevalence of use of traditional medicine was presented as percentage proportion and its 95% confidence interval. Categorical data were compared using Chi-square. The quantitative variables were first plotted to study the distribution. For normally distributed variables, we calculated the mean and standard deviation. For variables that were not distributed normally, we calculated the median and interquartile range. All tests were validated using two-sided P-value. If it was <0.05, we considered it as statistically significant.

## Results

We interviewed 1,000 persons, but 999 completed survey and one replied to less than 20% of the questions. So his response was excluded from final analysis.

The prevalence of traditional medication use for eye care was 21.9% (95% CI 19.3; 24.5). CAM utilization for eye care was statistically significantly more in Saudi females than males (P < 0.001). The variation of CAM utilization by type of eye ailment was statistically significant (P = 0.002) (Table 1).

**Table 1:** Complementary and alternative medicine utilization among Saudi adult population by determinants.

		Examined	Used traditional medication	Percentage proportion	95% confidence interval	Validation
All		1000	219	21.9	19.3; 24.5	
Gender	Male	362	46	12.7	9.3; 16.1	OR = 2.6 (95% CI 1.8; 3.6), P =0.00001
	Female	638	173	27.1	23.7; 30.6	
Age group	<20	84	13	15.5	7.7; 23.2	Chi square = 0.5
	21 to 40	546	123	22.5	19.0 ;26.0	Degree of freedom (DF) = 4
	41 to 60	276	62	22.5	17.5; 27.4	P = 0.5
	61 and more	94	21	22.3	13.9; 30.8	
Education level	Illiterate	147	31	21.1	14.5; 27.7	Chi square = 1.3
	School	351	69	19.7	15.5; 23.8	df = 4
	College	443	104	23.5	19.5; 27.4	P = 0.2
	Higher education	58	15	25.9	14.6; 37.1	
Residence	Central	617	141	22.9	19.5; 26.2	Chi square = 2.3
(in KSA)	Eastern	59	13	22	11.5; 32.6	df = 5
	Southern	165	38	23	16.6; 29.5	P = 0.1
	Western	75	16	21.3	12.1; 30.6	
	Northern	83	11	13.3	6.0; 20.5	
Type of systemic ailment	None	666	144	21.6	18.5; 24.7	Chi square = 3.5
	Diabetes	228	43	18.9	13.8; 23.9	df = 4
	Hypertension	46	9	19.6	8.1; 31.0	P = 0.06
	Other	59	23	39	26.5; 51.4	

Castor oil (49.3%), antimony (khohl alethmed) (40.6%), chamomile (19.6%), and green tea leaves (11.4%) were the most common medications used. Other traditional eye treatments included eye vitamins, faith healing (prayer, reciting the Quran, Zamzam water), cautery, cupping, and acupuncture.

Most participants applied the traditional medications using eye drops (54.2%), other methods were around the eye, orally and others with a percentage of (17.1%), (2%) and (40.2%), respectively. Other traditional eye treatments included: eye vitamins, faith healing (prayer, reciting Quran, Zamzam water), cautery, cupping, and acupuncture. Out of 67 responses, eye vitamins were mostly used (60.5%), followed by faith healing 13.2%. On enquiring about the reason behind using traditional medications the majority reported no effect of allopathic medications (71.1%), recommended by relatives and friends (13.3%). Few (3.6%) reported following physician’s advice. The most frequently reported source of knowing about traditional treatment was the internet (66.9%), followed by social media (13.3%). Thus, asking about whom they discussed with the idea of using traditional treatment for their eye (86.7%) reported not discussing it with anyone, and only (10.2%) discussed it with an Ophthalmologist. The duration of treatment for most participants lasted for a year or more (58%), and (24.6%) reported lasting for a few days only. Interestingly, nearly half of the participants (49.0%) reported improvement of their eye problem after using the traditional medication, (41.4%) did not notice any change, and (9.6%) worsened.

## Discussion

One in five people with eye problem uses CAM among the Saudi population. This CAM usage was more prevalent in females compared to males. Castor oil, Kohl and Chamomile were the most common CAM used in eyes. Use of eye drops as a medium for CAM delivery in eyes was common. Not satisfactory benefits of allopathic medications were the main cause of CAM usage. Two-thirds of the population came to know about CAM from the internet for eye ailments. Nearly half of the CAM users reported benefit in while one in ten noted worsening of symptoms after CAM usage.

This is perhaps the first study in the Saudi population giving insight about concurrent usage of CAM in eye diseases. It is lower than CAM usage for the other parts of the body. CAM in Saudi Arabia is very common and it ranged from 21.6% to 90.5% as reported in different studies [4,8,10,11]. CAM usage among Arabs could be due to the cultural and religious beliefs. In Islam, traditional medicine is referred to as the medicine of prophet. Muslims are encouraged to use some traditional medicines like honey, olive oil, blackseeds, zamzam water, and recitations from Quran to cure their health problems [11,12]. In this study, faith healing for eye ailments was uncommon (n = 13), whereas it was predominant in other studies [4,11]. This could explain the high prevalence of using CAM for other parts of the body compared to this study.

The prevalence of CAM use for eye care in our study 21.9% was marginally less than the prevalence in rural India (25%) as reported by Gupta et al. and in semi-urban communities in south Nigeria (48.7%) [15,17]. Such comparison should be done with caution as our study was held in the Capital; urban area of Saudi Arabia with easy and free access to eye services. The education level of the urban population could also be better than that in rural Saudi Arabia and CAM use was significantly less among educated than less educated [4,9,17].

In our study, CAM usage among the adult Saudi population did not significantly vary by age groups. CAM is commonly used in pediatric and old aged population [18,19]. Since our study focused on 20 years and older people with less than 10% of more than 60 years of age, rate of CAM usage could be an underestimate.

In this study, it was found that females are more likely to use traditional remedies for eye care than males (P < 0.001), It agrees with Smith et al. [20]. However, other studies did not report any gender significance [15,21]. An interpretation for this significance is the high percentage of using castor oil (49.3%), a popular remedy between women for eyelash growth, although there aren’t any scientific studies that prove eyelash growth after applying castor oil. Antimony (khohl alethmed), which is used by 40.6%, is another traditional remedy mostly used by women for cosmetics. 

Also, no significant association was found between education and the use of CAM in this study, it agrees with Carvalho et al. [22]. Whereas some studies revealed a significant association with higher education [7,10]. But the results do not match, with other studies that reported people with low education are more likely to use traditional medications [4,9,17]. These results variations conclude that cultural beliefs are strong indicators of using traditional medications, in addition, the perception that natural products are safe to use [4,23].

Different traditional remedies were reported like castor oil, antimony (khohl alethmed), chamomile, green tea, honey, lepidium sativum (Rashad) and many more. They are similar to the therapies used in general by the Saudi population [8]. A study done in Jordan revealed that green tea, aniseed, ginger, and chamomile were the most common herbs used by diabetic patients [24]. In Oman, the use of plant extract from ‘Calotropis procera’, honey, and wasam for treating ocular diseases lead to poor visual outcomes [25,26]. A local case report [15] reported severe ocular injury after self-administration of lepidium sativum (Rashad) which was used by eight participants in this study [14]. Herbs used between Arabs are different from those used in India, Brazil and Africa [15,22,26]. Therefore, it is important for practitioners to be aware of the most common traditional medications used in a certain country, in order to consider asking patients about them.

CAM utilization by type of eye ailment was also significant in this study. This significance is supported when comparing the prevalence of CAM use between patients with glaucoma (5.4%) to patients with inflammatory eye diseases (42%) in two different studies [20,27]. This could be attributed to the severity of eye symptoms, particularly in patients with uveitis [23].

Faith healing (prayer, reciting the Quran, Zamzam water) was uncommon (n = 13). Although the use of faith healing is very common in Saudi Arabia for other types of ailments with the prevalence of 82% and 74.8%, it’s not the same case for eye ailments, participants preferred the use of local medications [4,11]. 

The main reason for using CAM was due to no effect allopathic medication (71.1%). Jan et al. also reported the lack of definitive medical cure as a reason for the use of CAM [4]. This necessitates educating patients about the exact duration of treatment, availability of second-line treatments and most importantly if the ailment is curable or not.

Internet and social media were the commonest sources of knowing about traditional treatments (66.9%), (13.3%), respectively. This could be the reason why the majority (86.7%) did not discuss using CAM with anyone, and it reflects the amount of trust people have in the internet and social media. It is recommended that physicians involve themselves in social media to educate the community and correct health misconceptions.

Nearly half of our participants (49%) reported improvement of their eye ailment after using traditional treatments, similar positive effect was reported by patients with inflammatory eye diseases [20]. Local studies also reported that 41.4% of CAM users for dermatological conditions were satisfied by its effect and the majority are willing to continue using it in the future [11]. However, patient’s satisfaction for its effectiveness is not sufficient, since some of them could be using traditional medications along with allopathic medications. Although there are some clinical trials that reported some beneficial effects of traditional treatments, like honey for Vernal Keratoconjunctivitis, and castor oil for treating meibomian gland dysfunction [28,29]. Serious complications reported by other studies should be considered and known by both physicians and patients [13,14,25,30].

In this study, worsening of the eyes was reported by 9.6%, and the majority used CAM for a year or more. This may indicate that physicians don’t ask patients about traditional treatments routinely.

In our study, which is the first study done for CAM utilization for eye care in Saudi Arabia, with sufficient sample size, had limitations related to sampling bias. It was limited to people attending ophthalmology outpatient clinics at a governmental tertiary hospital and therefore we cannot generalize our results to the whole Saudi population. Also, our research did not study what were the common traditional treatments that were used for specific eye diseases, more studies should be done focusing on this point in order to perform future clinical trials.

## Conclusions

CAM usage for eye care is common and mostly used without discussing it with anyone. Therefore, it is recommended that physicians ask patients about traditional treatments routinely, and educate them about the risk of applying anything to their eyes without consulting an ophthalmologist. Also, awareness programs about using CAM, in general, should be introduced to the population through public campaigns and social media in order to minimize the prevalence and possible side effects. the traditional medicine among Arabs is unique and often does not match with that used in other civilizations. Thus the findings of the present study would be useful to all researchers who intend to study the pros and cons of concurrent usage of allopathy and traditional medications in eye care.
